# The Response of Beech (*Fagus sylvatica* L.) Populations to Climate in the Easternmost Sites of Its European Distribution

**DOI:** 10.3390/plants11233310

**Published:** 2022-11-30

**Authors:** Cătălin-Constantin Roibu, Ciprian Palaghianu, Viorica Nagavciuc, Monica Ionita, Victor Sfecla, Andrei Mursa, Alan Crivellaro, Marian-Ionut Stirbu, Mihai-Gabriel Cotos, Andrei Popa, Irina Sfecla, Ionel Popa

**Affiliations:** 1 Forest Biometrics Laboratory, Faculty of Forestry, “Ștefan cel Mare” University of Suceava, Universității Street, No. 13, 720229 Suceava, Romania; 2 Alfred Wegener Institute for Polar and Marine Research, Am Handelshafen Street No. 12, 27570 Bremerhaven, Germany; 3 Forestry and Plants Protection Department, Technical University of Moldova, Block 1, Stefan cel Mare si Sfant Boulevard 168, MD-2004 Chișinău, Moldova; 4 National Research and Development Institute for Silviculture “Marin Drăcea”, Calea Bucovinei No. 76bis, 725100 Câmpulung Moldovenesc, Romania; 5 Faculty of Silviculture and Forest Engineering, Transilvania University of Brașov, 500036 Brașov, Romania; 6 “Alexandru Ciubotaru” National Botanical Garden (Institute), 18 Padurii, str., MD-2002 Chisinau, Moldova; 7 Center of Mountain Economy, INCE-CE-MONT Vatra Dornei, Petreni Street No. 49, 725700 Vatra Dornei, Romania

**Keywords:** marginal beech population, vapor pressure deficit, dendrochronology, tree ring, growth

## Abstract

In the context of forecasted climate change scenarios, the growth of forest tree species at their distribution margin is crucial to adapt current forest management strategies. Analyses of beech (*Fagus sylvatica* L.) growth have shown high plasticity, but easternmost beech populations have been rarely studied. To describe the response of the marginal beech population to the climate in the far east sites of its distribution, we first compiled new tree ring width chronologies. Then we analyzed climate–growth relationships for three marginal beech populations in the Republic of Moldova. We observed a relatively high growth rate in the marginal populations compared to core distribution sites. Our analyses further revealed a distinct and significant response of beech growth to all climatic variables, assessing for the first time the relationship between growth and vapor pressure deficit (VPD) which described how plant growth responds to drought. These results highlight that accumulated water deficit is an essential limiting factor of beech growth in this region. In conclusion, beech growth in the easternmost marginal population is drought-limited, and the sensitivity to VPD will need to be considered in future studies to update the forest management of other economic and ecologically important species.

## 1. Introduction

Global and European environmental strategies and policies recognize climate change as the main threat to natural capital [1,2,3]. Extreme climatic events’ increased frequency and intensity are now a reality in Europe [4]. European forest ecosystems have been significantly affected in the last decades by increasing drought frequency and intensity coupled with wildfires and insect attacks, with hundreds of millions of Euros of economic and ecological services losses [5,6,7,8,9]. For the Republic of Moldova (Eastern Europe), both climate variability and simulation scenarios forecast an increase in drought intensity and frequency [10,11,12]. There, drought and water deficit are the main climatic threats to agricultural and forest systems, already generating high economic losses [13,14].

Beech (*Fagus sylvatica* L.) is one of Europe’s main tree forest species, growing in large areas under different environmental conditions, where it plays an essential economic and ecologic role [15]. Based on tree-ring width data on beech, many studies revealed its high growth sensitivity to drought [16,17]. Moreover, large-scale models indicate the growth decline of beech in the last decades, both in the core distribution region and at its edge-range [18,19,20]. Even though environmental changes are evident in all biomes, the challenges are significantly higher in the marginal population, where growing conditions are more restrictive [21]. Moreover, these marginal populations can have lower genetic diversity, limiting their adaptative capacity [22] or developing specific adaptations due to extreme environmental conditions [23,24,25,26].

Based on ecological theory, species distributions should be pushed at higher elevations or latitudes as a consequence of rising temperatures [27]. Different species distribution models suggest a future refuge of beech to higher elevations [28] or latitudes [29]. On the other hand, several dynamic forest models suggest no movement in beech distribution as precipitation is not predicted to change significantly [30,31,32]. While beech populations living at the edges of their natural distributions represent evidence that, in the past, in those regions, conditions were favorable, now, the status of marginal populations highlights a significant change in growing conditions [33].

Beech is considered a prospective species with a high adaptation capacity to increasing water deficit due to its high genotypic and phenotypic plasticity [23,24,25,26]. The marginal beech population offers essential information on the ability of this species to respond to drought and the genetic capacity to survive in an environment with low water availability [34]. Such information helps adapt current beech forest management strategies to optimal growing conditions, for which an increase in drought intensity and frequency is expected in the future. Providing sustainable forest management in high-risk climatic conditions requires updated knowledge of species adaptation and mitigation capacity to actual climate change. Here, we investigate the growth of three marginal beech populations in the Republic of Moldova (i) to develop reference tree ring chronologies for the easternmost beech distribution region, (ii) to assess the beech climate–growth relationship, and (iii) to analyze the spatio-temporal stability of beech response to climate. 

## 2. Results and Discussions

### 2.1. Chronology Description

**Figure 1:**
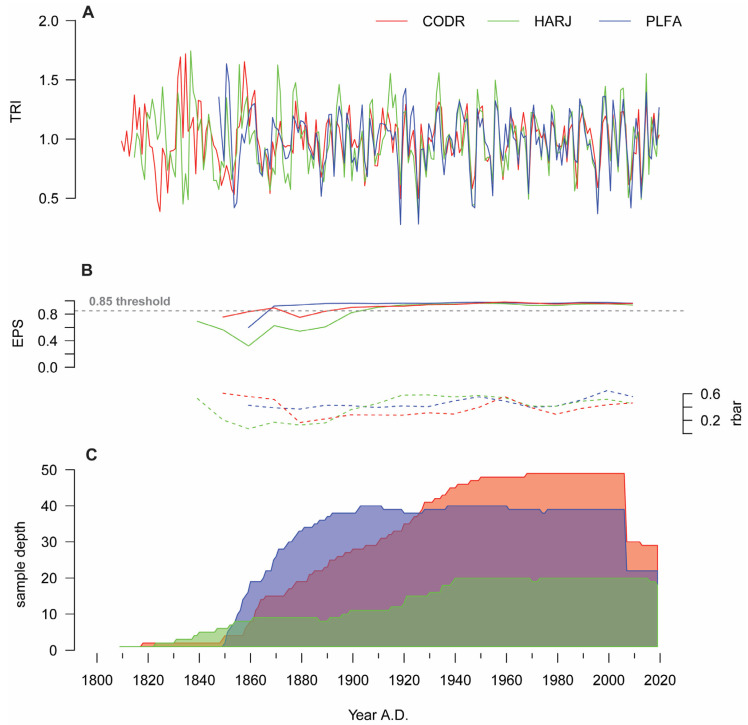
The marginal beech chronologies (**A**)—Tree-ring index chronologies, (**B**)—Chronologies’ statistics variation (EPS—continuous line and rbar—dashed line, (**C**)—Sample depth).

In this study, we developed three chronologies for marginal beech populations from the easternmost sites of its distribution (Figure 1). The mean tree age is barely uniform for all sites (126 ± 38 years), with maximum ages from 172 years (PLFA) up to 210 years (CODR). In terms of growth performance, the average growth rate of these marginal populations is 2.49 ± 0.62 mm year^−1^ and ranges from 2.23 mm year^−1^ (PLFA) to 2.68 mm year^−1^ (HARJ). The first-order autocorrelation is similar for all chronologies (0.58) and reflects a medium influence of the previous year’s environmental conditions on beech growth processes (Table 1). The chronology is likely robust from ~1880 AD to the present. Prior to this date, the chronology signal seems significantly biased by a reduction in sample size, and the remaining samples do not share growth variability. These findings are also supported by the high inter-series correlation values (rbar), and the expressed population signal, which exceeds the 0.85 threshold.

Besides these, the high signal-to-noise ratio (SNR) and mean sensitivity (MS) indicate the presence of a high climate signal within these beech populations (Table 1). Overall, these marginal beech chronologies’ statistical characteristics are similar to those of other range-edge chronologies [35,36,37,38]. 

Despite the beech growing at the easternmost edge of its distribution, the average growth rate is relatively high compared to other sites located at different distribution edges in Europe or the core distribution sites [27,39]. The present results contrast with other dendroecological studies, which have mentioned the beech growth declining in the core area [17,27], in high-altitude Central European forests [17], or other range-edge sites [35,40,41,42]. These faster accumulation rates can be the consequence of (i) CO_2_ fertilization in the last decades that induced growth stimulation [43,44]; (ii) the net effect of the large-scale atmospheric circulation (e.g., the jet stream latitude dipole) on beech radial growth and carbon uptake [45] and (iii) genetic and local environmental factors that act as drivers for locally adapted populations, especially in range-edge sites [27,39,46,47].

For all the analyzed chronologies, periodic reductions in growth occurred in 1887, 1892, 1896, 1901, 1904, 1925, 1946, 1995, 2007, 2008, 2012, 2013, and 2015. The years 1932–1933, 1943–1944, 1948, 1955, 1961, 1970, 1984, and 1988 are above mean growth. The obtained chronologies have a significant degree of synchronization, the lowest value of the dendrochronological statistics being identified between CODR and HARJ (tVBP = 14.4, Glk = 74) and the highest between HARJ and PLFA (tVBP = 20.0, Glk = 76). These higher synchronization and sensitivity values can result from the alternation between higher and lower growth rates, demonstrating that beech trees with accelerated growth are more reactive to extreme climatic events [27,46]. The periods with growth reductions highlight a high and increasing sensitivity to climatic variables, specifically to drought [27]. However, trees do not share the same variability in the innermost part of the chronologies, and the growing process seems to be more influenced by competitional processes and/or harvesting activities. In the Moldavian forests, the management until 1918 was orientated either to a coppice system characterized by clear cuts and short rotations (50–60 years) or a selection coppice system. These silvicultural activities can induce a higher variability in the beech growth and modify its marginally low sensitivity to environmental factors [48,49]. After the first world war, forest management was changed to a more intensive one, with lower harvesting intensity rates. Likewise, this new forest policy, valid until now, can explain the presence of beech trees older than 150 years. 

### 2.2. Beech Response to Climatic Conditions

**Figure 2:**
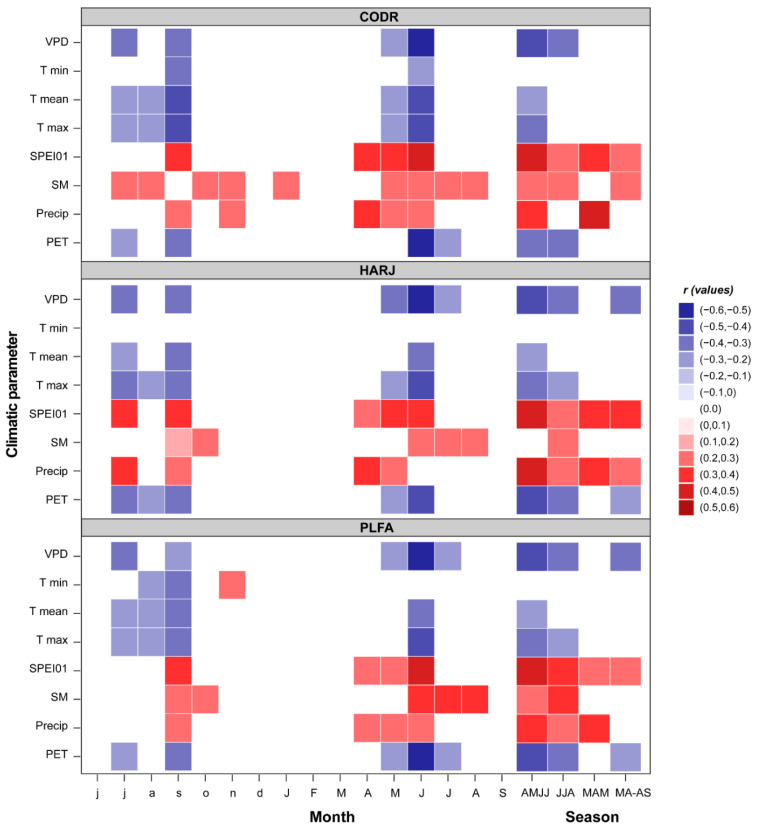
Climate growth relationship (color ramp represents significant bootstrap correlation) over the 1958–2019 period (uppercase months—current year, lower case months—previous year, VPD—vapor pressure deficit, Tmin—minimum temperature, Tmean—mean temperature, Tmax—Maximum Temperature, SPEI01—One-month Standardized Potential Evapotranspiration Index, SM—Soil Moisture, Precip—Precipitation, PET—Potential Evapotranspiration).

**Figure 3:**
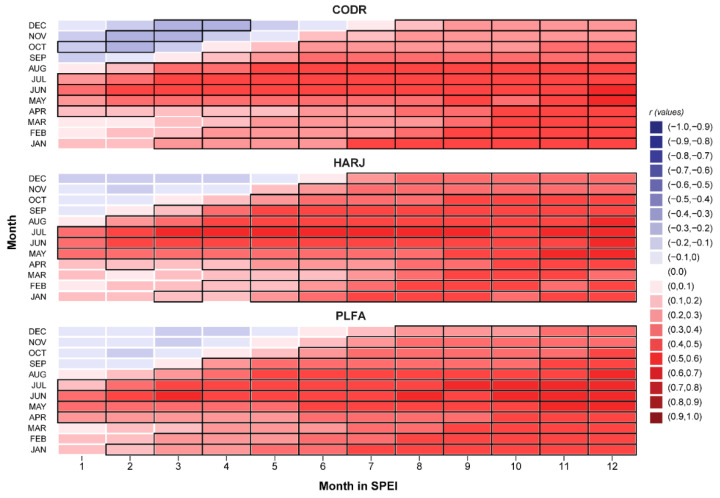
Correlation between growth indices and SPEI using different time scales (black line represents significant correlation).

Broadly, the easternmost edge beech populations reveal a distinct and significant response to all climatic variables (Figure 2). Spring to summer precipitations (r = 0.39) and SPEI1 (r = 0.45) expressed a stronger relationship with radial growth in all sites. Also, we found that beech has a high sensitivity to soil moisture during summer, with a maximum correlation of r = 0.35 for PLAI. In CODR and HARJ sites, where beech trees are growing on the bottom of valleys with higher moisture content, the SM has a reduced but still significant correlation with ring-width indices—TRI (r = 0.27). All beech sites showed a significant negative correlation with the maximum and mean temperature from April to July (r = −0.33, respectively r = −0.26) and insignificant correlations with the winter temperature. For the first time, it was assessed the relationship between beech TRI and VPD, showing that at marginal sites, the vapor pressure deficit plays an essential role in the beech growth mechanism. A high VPD during the first half of the vegetation season (April to July) induces low growth rates on beech (r = −0.45), while VPD in June has a more substantial influence (r = −0.52). In addition, beech has a similar reaction with PET (r = −0.40), having the highest significant correlation (r = −0.51) with June. The results also indicate that the previous year’s climatic conditions significantly influence beech growth. All chronologies are negatively correlated with the mean and the maximum temperatures from July to September of the previous year (r = −0.30), with September as the most critical month (r = −0.38). An exceptional situation is represented by the positive correlation between the minimum temperature of the previous November and TRI for the PLFA site (r = 0.27). VPD and PET of the prior year seem to have a weaker influence but are still significant compared to the winter and spring months of the current year (r = −0.36, respectively r = −0.31). Furthermore, beech positively responds to the hydroclimatic variables of the previous year (Precip, SPEI, and SM). Nevertheless, prolonged drought periods negatively influence marginal beech populations (Figure 3). The correlation between SPEI and TRI is positive and significant for all months for a time scale higher than 8 months. Also, this drought-influenced pattern is increasing in intensity from smaller time scales (1–4) to higher time scales (10–12). These results highlight that accumulated water deficit is an essential driver of beech growth in this region. 

Compared to the core or other rear-edge beech sites [18,20,35,46,50], the easternmost ones have a more consistent response to climate. Interestingly, although we analyzed marginal beech populations, our results contradict previous records showing that beech has poor climatic sensitivity in the Southern distribution edge [27,38]. However, our findings are in line with several other studies which have found that water availability is the main growth driver of beech in Europe [18,27,36,37,39,45,51,52]. Furthermore, the temperatures expressed a stronger relationship with beech growth [18,32,36,51,53,54], in some cases even higher than precipitation [28,39]. High temperatures play an essential role in drought severity [55], and “hotter droughts” increase evapotranspiration rates, influencing tree growth’s physiological mechanisms [56,57,58]. Specifically, an increase in evapotranspiration and the lack of water supply during the vegetation season may cause growth and vitality reductions directly affecting cell division and development, xylem embolism, phloem velocity, or indirectly photosynthesis activity, transpiration, and tree nutrition [32,56,59,60]. Nevertheless, higher evapotranspiration can also be an expression of the increased VPD. In previous physiological studies, VPD was identified as an essential driver of plant functioning and a significant contributor to climate-induced plant mortality [61,62]. Despite this, no dendroclimatological studies have previously assessed the relationship between VPD and beech TRI. In this study, we highlighted the negative effect of higher VPD on beech growth, which seems to act as a cumulative stress factor. Our results align with other experimental studies, which demonstrated that VPD limits tree growth, sometimes even before SM becomes a limiting factor [56].

Furthermore, the growth reduction is even more severe when VPD and other climatic factors (like SM or temperature) thresholds are exceeded [63]. This finding complements the above results and has a solid physiological background, considering higher VPD declines stomatal conductance and increases the transpiration rates, with higher risks of water loss, hydraulic failure, and growth reduction [53,61,62,64]. Another possible reason for this drought-induced growth pattern can be related to beech xylem architecture, a diffuse-porous wood exhibiting a significant legacy effect after drought and displaying susceptibility to water stress [64,65,66] and VPD [63]. Compared with other broadleaf species, beech displays an increased sensibility to drought [28,53,67], being more exposed and vulnerable to future climate change [18,56,63] and losing its competitive advantages in this context [28]. 

Even though beech is growing in a temperate climate with excessive influences, the winter and spring temperatures seem not to be limiting factors for growth in the easternmost sites. These findings contradict other studies from the coldest beech sites [68]. Nonetheless, in line with our study, the beech populations for the western and southern limits did not record the winter temperature signal either [27,46,53]. This could be argued based on the local population’s adaptation, the buds being resistant to frosts, or the increased frequency of mild winters and warm springs [46,59]. 

The beech response to the previous year’s climatic conditions (positive to water supply and negative to the temperature spectrum, VPD, and PET) is quite common, being identified in different sites across its European distribution [17,18,27,35,36,37,39,69]. The previous year’s summer temperatures and precipitation may trigger masting events the following season, reducing stem growth by diminishing the tree carbohydrates [39,70]. Alternatively, the observed negative influence of previous summer temperatures can be the direct effect of long-term droughts. During long dry periods, the beech trees tend to deplete soil water reserves more quickly, increasing the drought stress [56]. Similar patterns were found in the Bavaria region [53,71], Moldova, Eastern Romania [36,72], and even southern populations within the species distribution rear edge [35,42,52,73]. 

Furthermore, prolonged droughts induce metabolic imbalances by mobilizing soluble sugars (non-structural carbohydrates-NSC) from starch deposits to protect cells’ dehydration and defend the trees against pathogens [56]. Also, post-drought recovery and growth processes will further reduce the NSC levels. The increased frequency of prolonged droughts depletes these reserves, causing low growth rates in the current year. Additionally, drying soils could be a stress factor for beech roots, damaging them and reducing their hydraulic conductivity, which can also affect stem growth [74]. 

### 2.3. Spatio-Temporal Stability of the Climate–Growth Relationship

**Figure 4:**
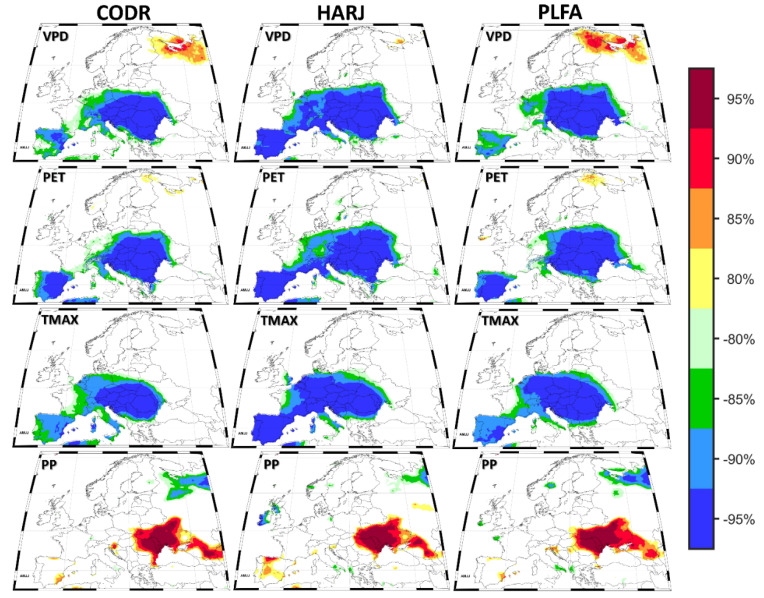
The stability map between the TRI for each site and the April–July (AMJJ) Vapor Pressure Deficit (VPD—first row); Potential Evapotranspiration PET—second row); maximum air temperature (Tmax—third row) and precipitation (Precip—fourth row). The regions where the correlation is stable, positive, and significant for at least 80% of windows are shaded with dark red (95%), red (90%), orange (85%), or yellow (80%). The corresponding regions where the correlation is stable but negative are shaded with dark blue (95%), blue (90%), green (85%), or light green (80%).

The relevance of the climate–growth relationship is determined by the strength of the correlation coefficient between tree growth and different climatic parameters and by the spatial correspondence between growth and climate. Thus, the spatial and temporal stationary of the climate signal needs to be tested individually for every study site, tree species, and climate parameter. For this study, we tested the spatio-temporal stationarity of the signal between the growth and the climate variables only for the April–July (AMJJ) period (Figure 4) since, for this period, we obtained the highest climate–growth correlation coefficients (Figure 2). The stability maps (Figure 4) indicate that the growth-related time series are significantly correlated with climate variables (e.g., VPD, PET, and Tmax) not only at the local scale (Figure 3) but also at the European level (Figure 4). The correlation presents a regional signal for all site locations only for precipitations. The correlation between all analyzed sites and VPD, PET, and Tmax climate variables is negative, stable in time, and significant over large areas covering the southern, central, and eastern parts of Europe. Opposite, the correlation between all analyzed sites and Precip is positive, stable in time, and significant over the eastern part of Europe, with a particular focus on the eastern part of Romania, the Republic of Moldova, and the western part of Ukraine. This large spatial extent of the correlations indicates that the signal captured by the tree ring width index of marginal beech populations from the easternmost sites at its European distribution is not only local but also part of a larger scale signal. The large spatial extent of the stable correlations could be an indicator that the large-scale factors (e.g., the large-scale atmospheric and oceanic circulation), which are influencing the local conditions over our analyzed region, might be the same as the ones influencing the growth in the south and south-eastern part of Europe (Figure 4).

**Figure 5:**
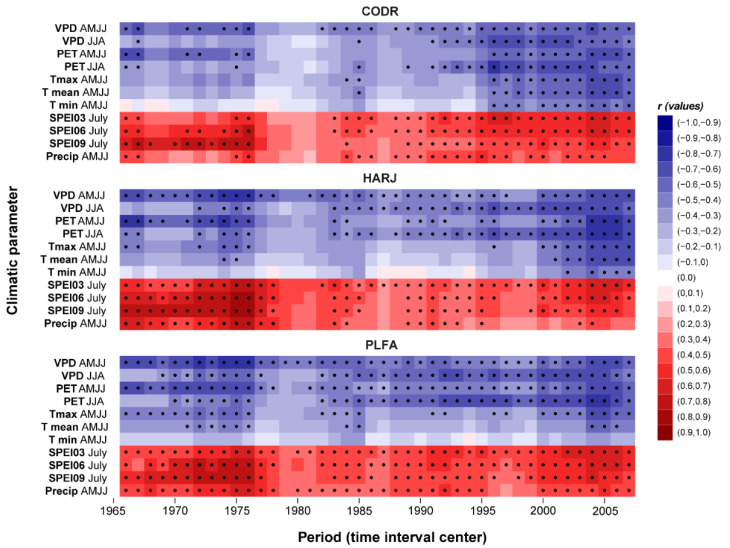
Temporal stability of climate growth relationship (dots represent significant correlation values, the abscise axis values represent the center of the 21-year time window).

In terms of temporal stability, drought remains the main driver of beech growth for the entire analyzed period and all the sites (Figure 5), with some exceptions. A weaker correlation was observed for the 1980s period, which could be explained by the cold and wet weather recorded from the 1970s to 1990s when the limiting factors probably decreased [57,58,75]. Alternatively, another possible reason for the climate signal weakening can be related to air pollution and acid rains in the second half of the 20th century, also known as “Waldsterben”. In line with our findings, different studies reported the same climate signal weakening in Central Europe [36,37,76,77,78,79]. Overall, a general pattern of increasing correlation between beech TRI and seasonal climatic parameters was identified, especially after the end of the 20th century. Nonetheless, no significant temporal shifts in the climate–growth relationship were noticed.

Additionally, the dendroclimatic response can be affected by uncertainties induced by the climate dataset’s quality or by the non-stationary tree growth response to climate. The exceptional climate variability may decouple or reduce the tree growth response to climate over time [80]. However, the increased correlation between the TRI and climatic factors highlighted in our study has strong climatic and physiological support. Over the last ~20 years, in the study area, an important change in aridity index variation has been observed, the area becoming more arid-prone (Figure S1).

The high temperature and evapotranspiration rates combined with the low water supply increased soil drying and compaction. These factors could modify nutrient and minerals uptakes inducing physiological dysfunctions in plants [61,63], which could further affect the temporal stability of the dendroclimatic pattern. Furthermore, considering the projected climate change scenarios, we expect a temperature and water deficit increase for the next 100 years [81,82,83]. In this context, VPD becomes an essential driver for beech growth and a significant contributor to tree mortality, considering this factor plays a crucial role in the increasing drought stress [61,63,84]. Nevertheless, it was demonstrated that genetic variations could play an essential role in the adaptive potential of the beech population to cope with these new climatic threats [63]. Consequently, new silvicultural management measures should be created and orientated to mitigate the adverse effects of climate on beech growth. 

## 3. Materials and Methods

### 3.1. Site Description

**Figure 6:**
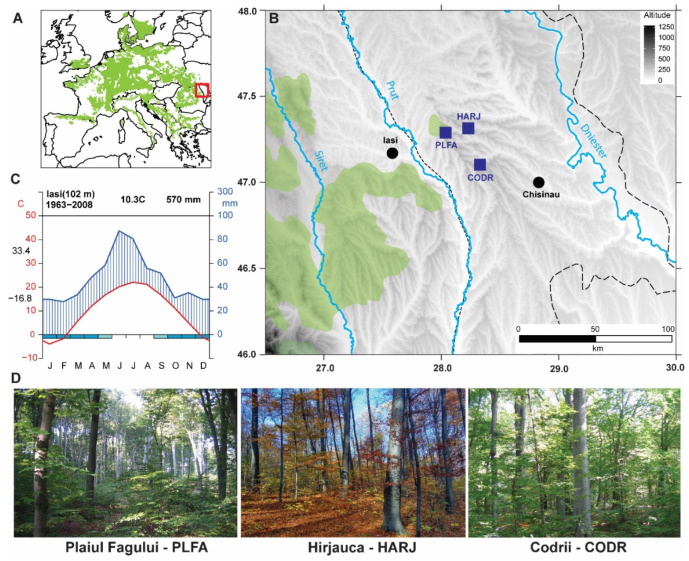
Study location (**A**)—Beech distribution in Europe [86], with a focus on the study area (red rectangle), (**B**)—Sampling areas locations—blue points represent the beech chronologies site, black dots—the main cities, dashed lines—national border, (**C**)—Walter climatic-diagram for Iasi weather station), (**D**)—representative images for each site).

The beech (*Fagus sylvatica* L.) forests from the Republic of Moldova are situated at their easternmost distribution in Europe (Figure 6, Table 2). In the Republic of Moldova, beech is scattered among three marginal populations, occupying only the valleys of the Central Moldavian Plateau [85]. The study area corresponds to a specific region of the Codrilor Plateau with wavy and fragmented terrain. In the shady slopes with brown luvic soil types, a mixture of oak and beech forest represents the predominant vegetation type.

The mean altitude is 240 m a.s.l., with a mean inclination of 10 degrees and slope aspect facing NE in all sites. The entire region has an excessively continental climate. The annual mean precipitation total over the last 60 years was 571.9 mm, and the mean temperature is 10.7 °C (with an amplitude ranging from −9.3 °C to +13 °C). For the warm period (April to November), the precipitation amount ranges from 189.2 mm (minimum) to 580.1 mm (maximum), which represents cc. 47%–86.7% of the annual precipitation. In these climatic conditions, beech faces functional growth limitations [87]. 

### 3.2. Chronology Development

A total number of 114 increment cores were sampled, one core per tree, with a Pressler borer from healthy and dominant trees in three separate field campaigns (2006, 2019, and 2020), and dried in plastic containers with ventilation slots. The cores were processed and measured in the Forest Biometrics Laboratory (biometrie.usv.ro (accessed on 25 November 2022).) and INCDS Marin Dracea Dendrochronology and wood anatomy Laboratory. After drying, surface preparation of increment cores was conducted by cutting plane surfaces using the WSL core microtome [88]. The tree ring boundary visibility was enhanced by filling the vessel lumina with chalk [89]. The samples were measured using a Lintab 6 system and TSAPwin software [90] with a precision of 0.001 mm. The measurement quality was visually checked in TSAPwin and statistically verified with COFECHA [91] using correlation analysis of 50-year intervals with 25-year overlaps. Each individual raw data series was transformed into a growth index series to remove the biological and potential non-climatically induced low- to medium-term growth trends. All tree rings were detrended using a cubic smoothing spline with a 50% frequency cut-off at 30 years in dplR library to maintain the high-frequency signal [92]. Tree-ring indices were computed as a ratio between raw and detrending functions, and the mean chronology was obtained using a bi-weight mean [75,93] with variance adjustment correction [94]. The temporal chronology strength and shared variance were assessed with the expressed population signal (EPS) parameter and inter-series correlation (rbar) using a 50 years window lagged by 25 years [95]. Additionally, standard dendrochronological statistics like mean sensitivity (MS), first-order autocorrelation (AC1), and signal-to-noise ratio (SNR) were computed for each chronology. The agreement between the chronologies was tested using the standard dendrochronological statistics: Gleichläufigkeit—glk [96] and the modified t value (tVBP) [97]. 

### 3.3. Climate–Growth Relationship

In the Republic of Moldova, there is a lack of reliable high-resolution climatic datasets. Therefore, monthly climatic gridded data were used, for the 1958–2019 period, from TerraClimate with 0.1 × 0.1° spatial resolution [98]. In this way, the dendroclimatic response of the marginal beech populations was evaluated using different climatic variables: total precipitation amount (Precip), mean air temperature (T mean), maximum air temperature (T max), minimum air temperature (T min), vapor pressure deficit (VPD), potential evapotranspiration (PET) and soil moisture (SM). Moreover, to assess the influence of water balance over the previous periods on the beech growth processes, we used the Standardized Potential Evapotranspiration Index—SPEI [99] monthly values for 1901–2019 for different accumulation periods (1–12 months). The multiscalar character of SPEI allows for highlighting the effect of long-term water deficit on beech growth. The monthly SPEI values for the different accumulation periods were downloaded from the global SPEI database (SPEIbase v. 2.7) [100]. In this study, we chose SPEI both for its multiscalar feature and for its capacity to incorporate the effect of temperature. The essential role of temperature on drought severity has been emphasized in different studies [101,102,103,104,105], and this effect was also evident in the extremely dry years of 2003, 2015, and 2018 in Europe [103,104,105]. The extremely high temperatures dramatically increased evapotranspiration throughout these extremely dry summers and exacerbated the summer drought stress. The aridity index (AI) has been captured by using the precipitation amount and evapotranspiration (Eto) from the TerraClimate database [98]. The AI is defined as Precip/Eto).

The climate–growth relationship was analyzed using monthly climatic variables for each chronology. The Pearson correlation coefficients were computed for the interval starting from the previous year June to the current year September, using the bootstrap method for significance testing from treeclim package [106]. The temporal stability of the relationship between the main climatic variables, which affects beech growth processes (mean, maximum, and minimum temperature, precipitation, potential evapotranspiration, vapor pressure deficit, SPEI (1 up to 12 months)), was tested using a moving time window of 21 years for April to July interval. All the correlations were presented as heat maps using the ggplot2 R package [107]. 

### 3.4. Stability Maps

Another significant aspect of the climate–growth relationship is the constancy (stationary) of the spatio-temporal signal, therefore spatio-temporal stability between the tree-ring index chronologies and climate variables was tested using the stability map approach [108]. This technique was initially applied to the seasonal forecast of the European rivers and Arctic Sea ice [108,109], but recently also demonstrated its usefulness in dendroclimatological studies [37,67,110,111]. The stability map method analyzes the spatio-temporal variability of the correlation between the TRI indices and climate variables with a 21-year moving window over the 1958–2020 period. For this study, the correlation is considered stable for those regions where the correlation between the TRI index and the climate gridded variable is significant above the 95% level for more than 80% of the 21-year moving windows. We performed these analyses between the TRI index (CODR, HARJ, and PLFA) and climate variables (VPD, PET, Tmax, and Precip) for the cumulation period from April to July (AMJJ). A detailed description of the methodology is given by Ionita [109]. Overall, the basic idea of this methodology is to identify regions with stable correlations (not changing over time) between the TRI index and the climatic gridded data (e.g., Precip) for different time lags.

## 4. Conclusions

Species growing conditions can significantly influence the climate–growth relationships. In the study sites in the Republic of Moldova, beech populations were scattered beyond the easternmost limit of their European distribution. At these sites, beech exhibits superior growth compared to its optimal core distribution, but the response to climatic factors is more substantial than in other marginal areas (e.g., the southern limit). Soil water supply during the growing season and the prolonged drought periods are the main limiting factors of beech growth in the study area. For the first time, we analyzed the effect of vapor pressure deficit (VPD) on the marginal beech population, showing that VPD plays a crucial role in beech growth and also widening the possibilities for more detailed studies regarding the VPD potential.

Our results provide consistent evidence that considering the spatio-temporal stability, the beech has a consistent response at the continental scale with most climatic factors, except for precipitation, which has a regional response. Furthermore, no significant temporal shifts in the climate–growth relationship were noticed.

In conclusion, there is a strong need for research combining VPD approaches with tree growth response to drought. This combination might offer better insights for marginal populations located at the edge of its distribution, considering their increased sensitivity to climate. Such analyses might help better understand the reaction of beech toward changing climate conditions and predict potential future climate-related modifications of species distribution. Since the studied area has become drier in the last two decades, deciphering the reaction of the marginal beech populations to climate is essential for adapting the current forest management strategies.

## Figures and Tables

**Table 1:** Marginal beech chronology’s descriptive statistics (MSL—mean sample length (years); MGR—mean growth rate (mm year^−1^); SD—Standard deviation; AC1—first order autocorrelation; rbar—inter-series correlation; EPS—expressed population signal; MS—mean sensitivity; SNR—the signal to noise ratio).

Site	Raw Data				Detrended Data			
	Time Span	MSL ± SD	MGR ± SD	AC1	rbar	EPS	MS	SNR
CODR	1809–2019	121 ± 30	2.56 ± 0.53	0.58	0.364	0.966	0.33	28.1
HARJ	1814–2019	122 ± 49	2.68 ± 0.61	0.57	0.453	0.948	0.42	18.22
PLFA	1847–2019	135 ± 35	2.23 ± 0.74	0.58	0.452	0.972	0.35	34.64

**Table 2:** Study site locations and characteristics.

Code	Location	Number of Samples	Latitude (Degrees)	Longitude (Degrees)	Altitude (m a.s.l)
CODR	Codrii	49	47.10 N	28.32 E	244
HARJ	Hîrjauca	22	47.31 N	28.23 E	250
PLFA	Plaiul Fagului	43	47.28 N	28.02 E	225

## Data Availability

The datasets analyzed during the current study are available from the corresponding author upon reasonable request.

## Supplementary Materials

The following supporting information can be downloaded at: https://www.mdpi.com/article/10.3390/plants11233310/s1, Figure S1: Aridity Index (AI) over different climatological periods: (a) 1961–1990; (b) 1971–2000; (c) 1981–2010 and (d) 1991–2020.
