# Supplementary figures and images for: The Response of Beech (Fagus sylvatica L.) Populations to Climate in the Easternmost Sites of Its European Distribution
Source: Plants (Basel). 2022 Nov 30;11(23):3310. doi: 10.3390/plants11233310 (PMC9738208; doi:10.3390/plants11233310)

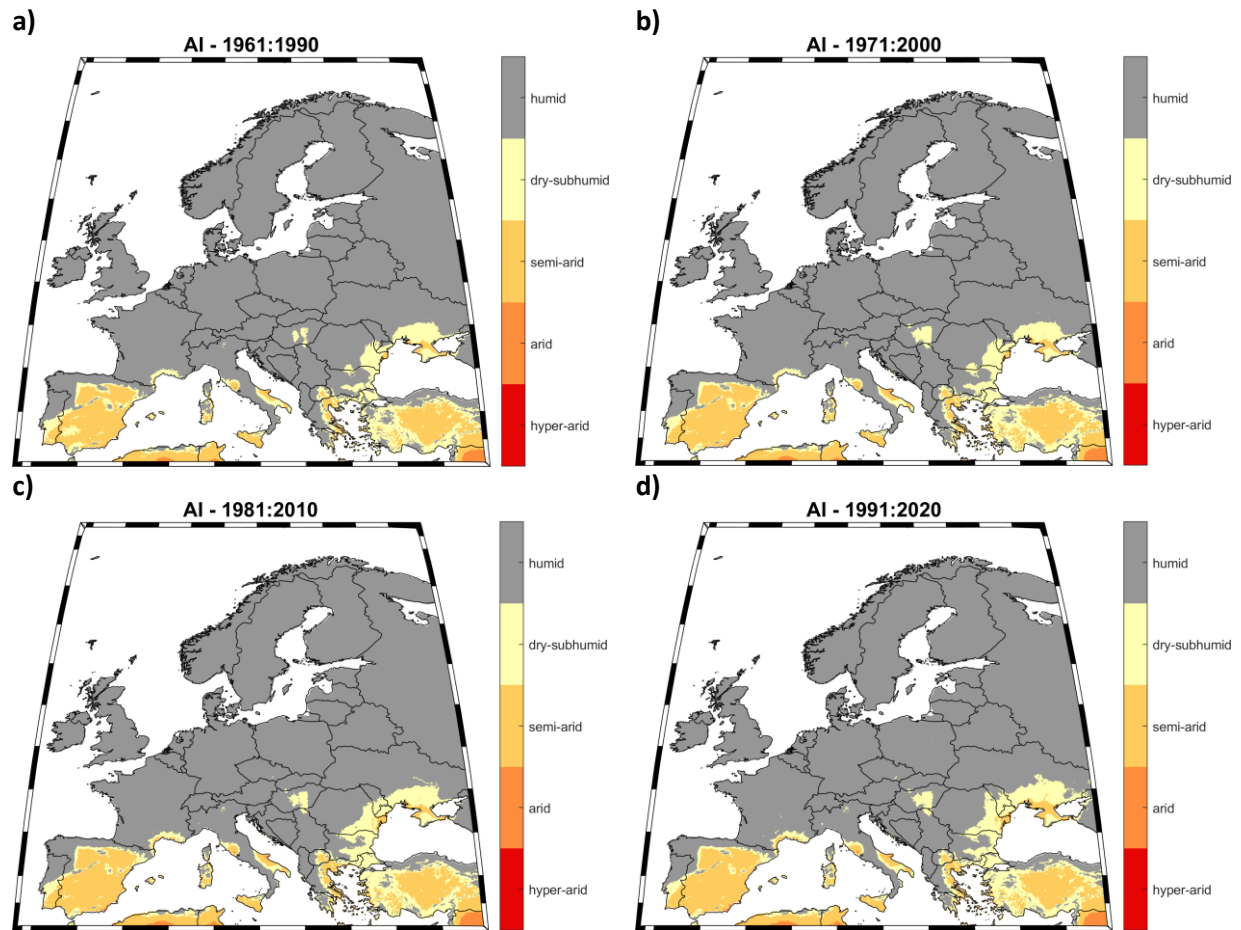

**Figure S1.** Aridity Index (AI) over different climatological periods: a) 1961 – 1990; b) 1971 – 2000; c) 1981 – 2010 and d) 1991-2020

Supplement: Supplementary file 1 [file plants-11-03310-s001.zip › plants-2065113-supplementary.pdf]
